# Tamarixetin Suppresses Colorectal Cancer Progression by Targeting DPP7‐Mediated WNT3A/β‐Catenin Signalling Pathway

**DOI:** 10.1111/jcmm.70787

**Published:** 2025-08-22

**Authors:** Peng Ouyang, Jin Gong, Jinlin Nie, Sridhar Kandala, Yangdong Shi, Yao Tian, Zhijing Zhang, Sifu Fang, Fan Pan, Lin Qiu, Zhen Bao

**Affiliations:** ^1^ Department of General Surgery The First Affiliated Hospital of Jinan University Guangzhou Guangdong China; ^2^ Department of Hepatobiliary Surgery First Affiliated Hospital of Gannan Medical University Ganzhou Jiangxi China; ^3^ Department of Gastrointestinal Surgery The Affiliated Guangdong Second Provincial General Hospital of Jinan University Guangzhou Guangdong China; ^4^ Medical Oncology Erasmus Medical Center Rotterdam the Netherlands; ^5^ The Affiliated Huizhou Hospital Guangzhou Medical University Huizhou Guangdong China; ^6^ State Key Laboratory of Respiratory Disease, Department of Otolaryngology–Head and Neck Surgery, Laboratory of ENT‐HNS Disease, First Affiliated Hospital Guangzhou Medical University Guangzhou Guangdong China; ^7^ Medical Imaging Center The First Affiliated Hospital of Jinan University Guangzhou Guangdong China

**Keywords:** anticancer therapy, colorectal cancer, DPP7, Tamarixetin, WNT3A/β‐catenin Signalling pathway

## Abstract

Colorectal cancer (CRC) patients have had limited benefits from conventional chemotherapy, highlighting the need for improved therapeutic strategies. Natural compounds have emerged as promising alternatives due to their potent anti‐cancer properties and reduced side effects. Tamarixetin is an O‐methylated flavonol derived from *Azadirachta indica* , but its potential and clinical utility to suppress CRC progression remain unknown. To figure out the underlying mechanism, the inhibitory effects of Tamarixetin on CRC were evaluated by in vitro assays; the validation of Tamarixetin‐mediated tumour suppression was performed with CRC xenografts and patient‐derived organoids. Our results demonstrated that Tamarixetin significantly reduced the proliferation of CRC cells (HT‐29 and HCT‐116) in a dose‐dependent manner, with minimal effects on normal colonic epithelial cells (NCM460). Furthermore, Tamarixetin inhibited proliferation, migration, and invasion of CRC cells, leading to reduced xenograft tumour growth and sensitising CRC to Oxaliplatin. Mechanistically, The expression and protein levels of DPP7 in CRC cells were suppressed by Tamarixetin, which lead to the downregulation of WNT3A/β‐catenin signalling pathway. This study highlights Tamarixetin as a promising natural compound for CRC treatment by interfering with DPP7‐mediated WNT3A/β‐catenin signalling pathway. These findings provide a novel therapeutic strategy to improve outcomes of CRC.

## 1 Introduction

Colorectal cancer (CRC) is one of the predominant malignancies within the digestive tract, significantly jeopardising global health [1]. Traditional therapeutic strategies, including surgical intervention, radiotherapy, and chemotherapy, fail to achieve a definitive cure for CRC in specific instances due to their limited efficacy [2]. Notably, the propensity of CRC cells to acquire resistance against conventional chemotherapeutic agents exacerbates the challenges during treatment [3]. Consequently, there is an urgent and crucial need to discover and develop novel therapeutics.

A distinct value of natural compounds has emerged for alternative anti‐tumour therapies [4]. These compounds show lethality in cancer cells with fewer side effects in comparison to conventional treatments [5]. Tamarixetin, predominantly derived from the leaves of *Azadirachta indica* , is a naturally occurring O‐methylated flavonol with diverse biological activities [6]. Many pieces of evidence show that Tamarixetin is used for cardioprotection [7], gastric safeguarding [8] and applied in anti‐inflammatory [9] and antitumor treatment [10]. Notably, Tamarixetin suppresses liver cancer progression through its impact on mitochondrial apoptosis and exerts inhibitory effects on human leukaemia cells by the induction of apoptosis and cell cycle arrest in the G2/M phase [6, 10]. However, the therapeutic effects of Tamarixetin in colorectal cancers remain to be thoroughly investigated.

Dipeptidyl Peptidase 7 (DPP7) is highly associated with tumour progression, and the knockdown of DPP7 increases apoptosis in tumour cells [11, 12]. On the other hand, DPP7 enhances the viability of tumour cells by promoting the progression of the cell cycle and inhibiting the DNA damage response [13]. On this basis, DPP7 targeting may improve treatment for a variety of cancer types, including CRC. Nevertheless, no DPP7 inhibitors have been developed, and new strategies are needed to specifically target the CRC overexpressing DPP7.

In this study, we examined this novel natural compound, Tamarixetin, and investigated the efficacy and safety of Tamarixetin in the treatment of CRC cancer. Our findings will further elucidate the mechanisms by which targeting DPP7 interrupts WNT/β‐catenin signalling, providing a theoretical basis for the application of Tamarixetin in CRC cancer.

## 2 Materials and Methods

### 2.1 Cell Culture

The CRC cell lines NCM460, HT‐29, and HCT‐116 were procured from the National Collection of Authenticated Cell Cultures. These cell lines were maintained in RPMI 1640/Dulbecco's Modified Eagle Medium (DMEM), enriched with 10% fetal bovine serum (FBS) and 1% penicillin–streptomycin, at a constant temperature of 37°C in an atmosphere containing 5% CO_2_.

### 2.2 Cell Viability Assay

Cells were seeded in 96‐well plates at a density of 5000 cells per well and cultured for 24 h, followed by treatment with varying concentrations of Tamarixetin (0, 50, 100, 150 μM) (Topscience, Shanghai, China). Cell viability was assessed 24 h post‐treatment using the MTT assay (Sigma‐Aldrich, USA), with absorbance measured at 490 nm. The half‐maximal inhibitory concentration (IC50) values were determined using GraphPad Prism version 9.5.0 software.

### 2.3 Drug Sensitivity Test of Organoids

Freshly collected CRC patient‐derived specimens were cultured in matrigel for organoids. After one week of culture and colony expansion, organoids were digested and inoculated with about 4000 live cells per well. After cells and collagen were fully diluted and mixed at a ratio of 1:1.3, the cells were incubated with 60 μL per well of CRC cancer medium. After the organoids expanded, organoids were treated with Oxaliplatin for 3 days. The area of organoids was measured.

### 2.4 Production of Lentiviruses

In this research, lentiviral vectors provided by GenePharma (Shanghai, China) were employed for transfection. The experimental design comprised groups for DPP7 overexpression (OE‐DPP7) and its respective negative control (OE‐NC), along with groups for DPP7 silencing (sh‐DPP7) and their corresponding negative control (sh‐NC). Stable colorectal cancer cell lines were established through selection with 400 μg/mL neomycin.

### 2.5 Wound Healing Assay

Cells were cultured in 6‐well plates and upon reaching 95% confluency, sterile pipette tips were employed to create linear scratch wounds. Following treatment with Tamarixetin (0, 50, 100 and 150 μM) or Box5 (100 μM), scratch wound conditions were documented at 0 and 48 h, respectively.

### 2.6 Transwell Migration and Invasion Assay

To assess cell migration and invasion, the Transwell assay was employed. Specifically, for invasion analysis, 2.5 × 10^4^ CRC cells were seeded in an upper chamber, coated with a 20‐fold dilution of Matrigel, containing 200 μL of serum‐free DMEM/1640. The lower compartment was supplemented with 500 μL of DMEM/1640, enriched with varying concentrations of Tamarixetin (0, 50, 100, 150 μM) or Box5 (100 μM). Following a 24‐h incubation period, cells that did not invade through the Matrigel were gently removed from the inner side of the upper chamber using a cotton swab. The cells that successfully invaded through the Matrigel to the outer side of the upper chamber were fixed in 4% paraformaldehyde for 10 min, subsequently stained with crystal violet, and imaged with a light microscope. The protocol for migration determination mirrored that of the invasion assay, with the sole distinction being the absence of Matrigel coating in the upper chamber.

### 2.7 EdU (5‐Ethynyl‐2′‐Deoxyuridine) Assay

In the proliferation assay, cells were evenly seeded into a 96‐well plate at a density of 10^4^ cells per well and incubated overnight in a CO_2_ incubator to facilitate adhesion. Following incubation, staining was performed using the Cell‐Light EdU Apollo567 In Vitro Kit (RiboBio, Guangzhou, China) as per the manufacturer's protocol.

### 2.8 Real‐Time Quantitative PCR

After treatment with Tamarixetin (0, 50, 100, 150 μM) for 24 h, cells were lysed using Trizol reagent for total RNA extraction. cDNA was synthesised from 1 μg of total RNA using the Prime Script RT reagent kit. The synthesised cDNA was then combined with SYBR Premix Ex Tap reagent kit and PCR forward and reverse primers, and RT‐qPCR experiments were conducted on a PCR system (Roche, Indianapolis, IN, USA). Data obtained were analysed using the 2‐ΔΔCt method. The sequences of the gene primers are presented in Table S1.

### 2.9 Western Blot

Cells were treated with Tamarixetin at concentrations of 0, 50, 100 and 150 μM and lysed in RIPA buffer on ice for 30 min. Nuclear and cytoplasmic proteins were subsequently extracted according to the protocol of the Nuclear and Cytoplasmic Protein Extraction Kit (Beyotime, China). Protein concentrations were determined using the Pierce BCA Protein Assay Kit. Each cell sample, containing 30 μg of protein mixed with 5× loading buffer, was loaded onto 8%–12% SDS‐PAGE gels for separation. The gels were then transferred to PVDF membranes (Millipore, Darmstadt, Germany), which were blocked with 5% skim milk for 1 h and incubated overnight at 4°C with primary antibodies. After washing three times with TBST, the membranes were incubated with HRP‐conjugated secondary antibodies at room temperature for 1 h followed by three washes. Protein bands were visualised using the Super Signal West Pico Chemiluminescent Substrate and imaged with an automatic chemiluminescence imaging analysis system (Tanon 5200, Shanghai, China). The primary antibodies used were against PCNA (#13110), Bcl‐2 (#15071), Bax (#89477), E‐cadherin (#3195), N‐cadherin (#13116), β‐catenin (#19807), and β‐Actin (#3700) sourced from Cell Signaling Technology (Boston, USA), with vimentin (AF#7013) from Affinity (Melbourne, Australia). Secondary antibodies (#AP132P and #AP124P) were obtained from Millipore Corporation (Massachusetts, USA).

### 2.10 Xenograft Tumour Model of CRC Cells

All animal procedures were carried out in specific pathogen‐free (SPF) conditions with the approval of the Animal Ethics Committee of Jinan University (Approval No. IACUC‐20240322‐06), in compliance with the animal care guidelines established by the Laboratory Animal Center of Jinan University. Sixteen male BALB/C nude mice were procured from the Laboratory Animal Center of Southern Medical University (Guangzhou, China). The mice were provided with sterile food and water and maintained in a controlled environment at a consistent temperature and humidity, subject to a 12‐h light/dark cycle. Subcutaneous injections of 1 × 10^6^ HCT‐116/HT‐29 cells were administered to 4‐week‐old nude mice. After four days, the mice were randomly allocated to either a control group (receiving tamarixetin at 0 mg/kg; 0.2 mL per mouse via intraperitoneal injection every other day for a duration of 14 days) or a tamarixetin treatment group (administered tamarixetin at 25 mg/kg; 0.2 mL per mouse via intraperitoneal injection every other day for 14 days). Tumour sizes were measured bi‐daily using callipers, and volumes calculated using the formula: volume = (length × width^2^)/2. Following a 3‐week treatment period, mice were euthanized, and tumours were harvested. Tumour specimens were bifurcated, with one half fixed in 4% formaldehyde overnight for subsequent Haematoxylin and Eosin (H&E) staining, and the other half preserved at −80°C for future analysis. The stained tissues were examined under an Olympus microscope (Tokyo, Japan).

### 2.11 Haematoxylin–Eosin (H&E) Staining

Tissues harvested from xenograft tumours were fixed in 4% paraformaldehyde, embedded in paraffin, and sectioned to a thickness of 5 μm. These sections were deparaffinised using xylene and subsequently rehydrated through a graded ethanol series (100%, 90%, 80%, 70%). Following rehydration, sections were stained with haematoxylin and eosin in accordance with established protocols. Photographs of the H&E‐stained sections were captured using a light microscope for further analysis.

### 2.12 Isolating and Culturing of Patient‐Derived Organoids (PDOs)

The use of patient samples was allowed by The First Affiliated Hospital of Jinan University IRB (Ethics Approval # KY‐2023‐325). CRC tissues isolated from primary tumour were minced into fragments, diced into small pieces, and digested for 30 min at 37°C with orbital shaking in digestion buffer consisting of Advanced DMEM/F12 supplemented with 2.5% fetal bovine serum, 1% penicillin/streptomycin, 75 U/mL type IX collagenase, and 1.25 mg/mL type II dispase. Then, basal medium containing 10 mM HEPES and 2 mM GlutaMAX was added, and the mixture was passed through a 100 μm cell strainer to remove large debris. Cells were pelleted at 1000 rpm (≈200 × g) for 3 min, resuspended in basal medium, and centrifuged again at 1000 rpm. This wash cycle was repeated twice to eliminate residual debris and enzymes. Tumour cells were finally suspended in Basement Membrane Extract (BME) and plated as 40 μL domes per well of a 24‐well plate. BME was polymerised for 20 min at 37°C, 5% CO₂, then overlaid with 500 μL complete human colorectal cancer (CRC) organoid medium: basal medium plus 20% R‐spondin‐1 conditioned medium, 100 ng/mL recombinant mouse Noggin, 1 × B27 supplement, 1.25 mM N‐acetylcysteine, 10 mM nicotinamide, 50 ng/mL EGF, 10 nM gastrin, 500 nM A83‐01, 5 μM SB202190, 10 nM prostaglandin E₂ and 100 μg/mL Primocin. Medium was refreshed every 2 days.

After reaching high density, BME domes were mechanically disrupted with a pipette and organoids were collected into a tube. After centrifugation at 1000 rpm for 3 min, the supernatant was removed. Organoids were incubated with 1 × TrypLE Express for ~5 min at 37°C, then triturated into small cell clusters. Clusters were washed with HBSS, pelleted at 1200 rpm for 5 min at 4°C, resuspended in fresh BME and re‐seeded at the appropriate ratio. Mycoplasma contamination was routinely tested by nested PCR. Organoids were banked in FBS containing 10% DMSO.

### 2.13 TCGA Database Analysis

Dataset and Acquisition: Gene expression and clinical data for colon adenocarcinoma (COAD) were downloaded from The Cancer Genome Atlas (TCGA) via the Genomic Data Commons (GDC) portal (https://portal.gdc.cancer.gov/). We selected the TCGA‐COAD project and retrieved raw count matrices and corresponding clinical annotations for all available tumour and adjacent normal samples.

Data Format: Expression data were in gene‐level raw counts (ENSEMBL gene identifiers). Clinical data included patient demographics, survival time, and vital status.

Preprocessing and Normalisation: Raw counts were imported into R (v4.2.0) and processed using the DESeq2 package (v1.36.0). Genes with zero counts in > 50% of samples were filtered out. Count data were normalised by the median‐of‐ratios method implemented in DESeq2, yielding variance‐stabilised expression values for downstream analyses.

Differential Expression Analysis: We performed differential expression between tumour and normal tissues using the DESeq2 Wald test. Adjusted *p* values (Benjamini–Hochberg correction) < 0.05 were considered significant, and log_2_ fold‐change thresholds of |FC| ≥ 1 were additionally applied to define differentially expressed genes (DEGs).

Prognostic Gene Screening: To identify genes associated with overall survival, univariate Cox proportional hazards regression was conducted for each DEG using the survival R package (v3.3‐1). Genes with Cox model Benjamini–Hochberg–adjusted *p* values < 0.05 were deemed prognostic.

Class Label Assignment for Survival Analysis: For each prognostic gene, patients were ranked by its normalised expression. The lower 50th percentile was assigned to the “low‐expression” group and the upper 50th percentile to the “high‐expression” group.

Survival and Statistical Cut‐Offs: Kaplan–Meier survival curves were generated and compared by log‐rank test. Hazard ratios with 95% confidence intervals were reported. Throughout all analyses, statistical significance was defined as adjusted *p* value < 0.05.

### 2.14 Statistical Analysis

All in vitro experiments were conducted across three independent replicates. Data are presented as mean ± standard deviation (SD) and were statistically evaluated using GraphPad Prism version 9.5.0. Differences among three or more groups were analysed via one‐way ANOVA, while comparisons between two groups were assessed using Tukey's post hoc tests. A *p* value of less than 0.05 was considered statistically significant.

## 3 Results

### 3.1 Tamarixetin Inhibits the Proliferation, Invasion and Migration of CRC Cells

**FIGURE 1:**
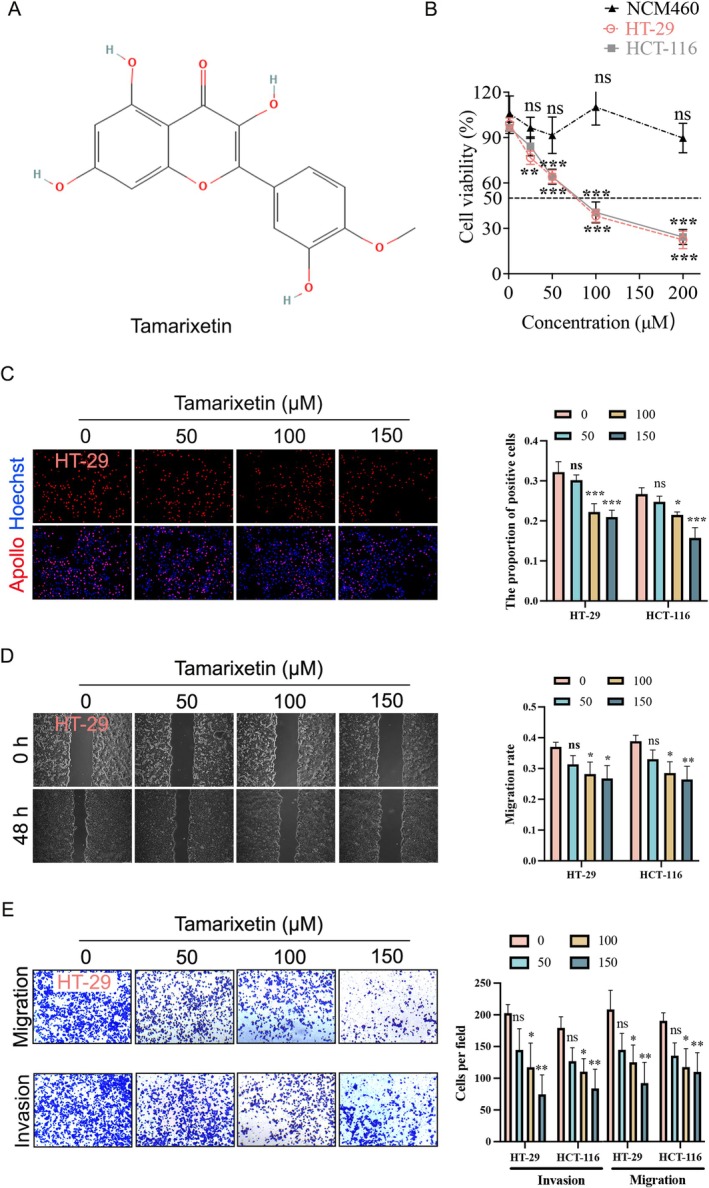
Tamarixetin inhibits the proliferation, invasion, and migration of CRC cells. (A) Chemical structure of Tamarixetin. (B) NCM460, HT‐29 and HCT‐116 cells were treated with Tamarixetin (0, 50, 100 and 150 μM) for 24 h, and then cell viability was determined by MTT assay, *N* = 5. (C) (Left) HT‐29 and HCT‐116 cells were treated with Tamarixetin (0, 50, 100 and 150 μM) for 24 h, and then EdU assay was used to detect cell proliferation. (Right) Corresponding statistical bar graph for EdU assay, *N* = 3. (D) Representative images and quantitative analysis of CRC cell migration based on wound healing assay. (E) The effect of Tamarixetin on the migration and invasion of CRC cells, *N* = 3. *p < 0.05; **p < 0.01; ***p < 0.001 versus non‐treated cells.

The chemical structure of Tamarixetin illustrated in Figure 1A. CRC cells (HT‐29 and HCT‐116), which have different APC backgrounds, showed a dose‐dependent reduction of viability after 24 h of treatment with Tamarixetin (Figure 1B). The IC50 values of these cells upon treatment with Tamarixetin were 72.04 μM (HT‐29, 95% CI: 66.19–78.41 μM) and 79.19 μM (HCT‐116, 95% CI: 72.02–87.22 μM), respectively (Figure 1B). Notably, Tamarixetin exhibited no significant cytotoxic effects on normal human colonic mucosal epithelial cells (NCM460) (Figure 1B). EdU assay showed a significant decrease in the proliferation of CRC cells after treatment (100 μM and 150 μM Tamarixetin), compared to control cells (0 μM Tamarixetin) (Figures 1C and S1A). Furthermore, Tamarixetin treatment prevented CRC cell migration as shown by wound‐healing assays and transwell assays (Figures 1D,E and S1B,C). Additionally, Tamarixetin also reduced CRC cell invasion in a dose‐dependent manner (Figures 1E and S1C). These results suggest that Tamarixetin treatment has an inhibitory effect on CRC cell proliferation and migration.

### 3.2 Tamarixetin Prevents the Progression of CRC Xenografts

**FIGURE 2:**
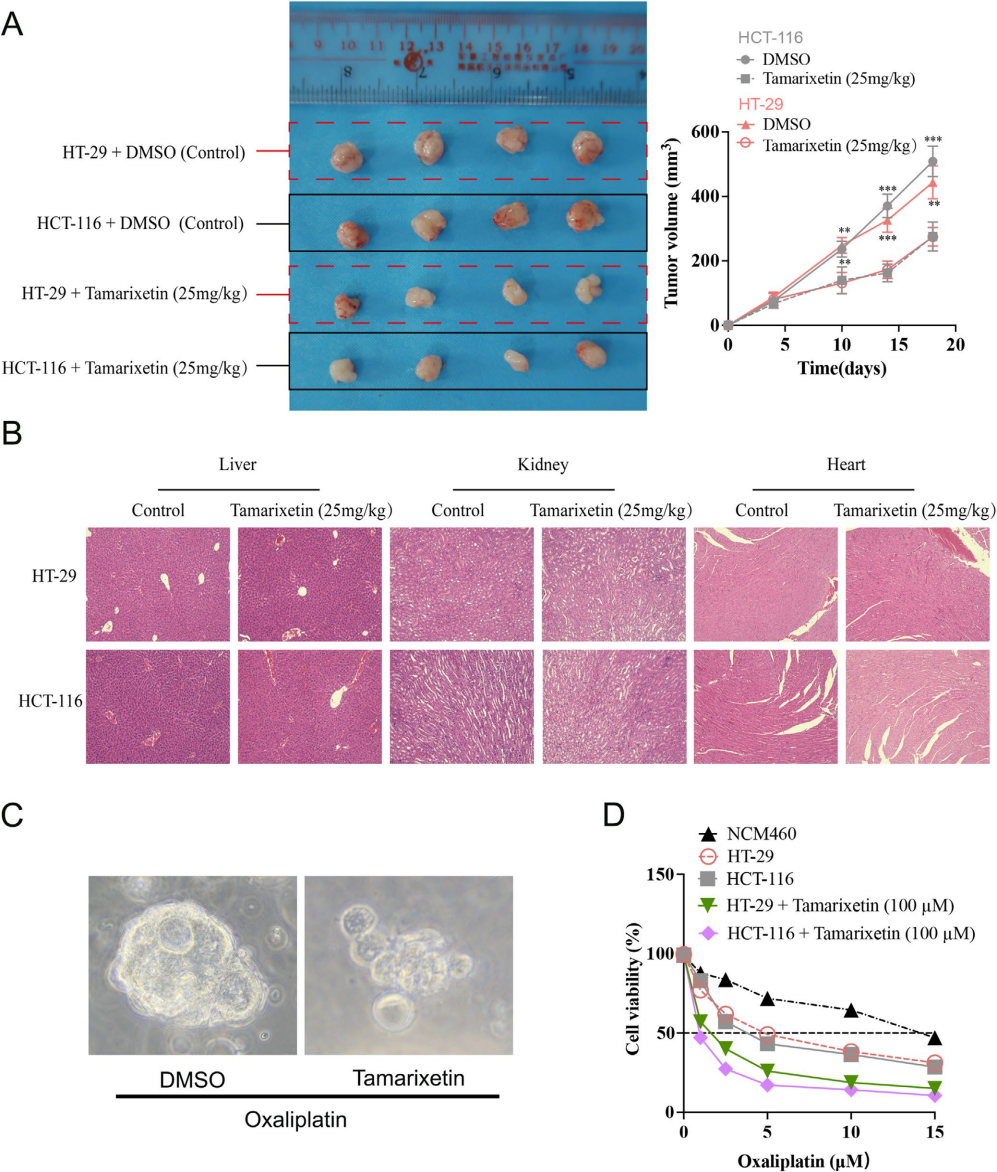
Tamarixetin inhibits the proliferation, invasion, migration and promotes apoptosis of CRC cells. (A) (Left) Nude mice bearing HT‐29 and HCT‐116 tumour of control and Tamarixetin‐treated groups. (Right) Tumour volume was monitored to measure the tumour growth in vivo. (B) Tamarixetin showed no significant effects on organ functions including liver, kidney, and heart after 18‐day administration analysing by HE staining, *N* = 3. *p < 0.05; **p < 0.01; ***p < 0.001 versus non‐treated cells. (C) The representative image of CRC PDOs under the treatment of Tamarixetin (100 μM) and Oxaliplatin (5 μM) for 24 h. (D) MTT assay was used to detect the cell viability of each treatment group. *p < 0.05; **p < 0.01; ***p < 0.001.

To further investigate the effect of Tamarixetin in vivo, mice with CRC xenografts were treated with 25 mg/kg Tamarixetin by intraperitoneal injection. These mice exhibited significantly smaller subcutaneous tumours after treatment (Figure 2A). H&E staining of the liver, kidney, and heart tissues from both the experimental and control groups revealed no obvious cellular damage (Figure 2B). Oxaliplatin is the most widely used first‐line chemotherapy drug for CRC. To determine whether Tamarixetin can sensitise CRC cells to Oxaliplatin, we generated organoids with relapse CRC samples from patients after continuous administration of Oxaliplatin and evaluated the effects of Tamarixetin on Oxaliplatin sensitivity in these organoids. Tamarixetin significantly enhanced the sensitivity of CRC organoids to Oxaliplatin, as evidenced by lower IC50 values compared to Oxaliplatin‐treated cells (Figure 2C,D). Collectively, these data indicate that Tamarixetin specifically targets CRC cells and enhances Oxaliplatin sensitivity in patient‐derived CRC organoids.

### 3.3 Tamarixetin Decreases DPP7 Expression in CRC Cells

**FIGURE 3:**
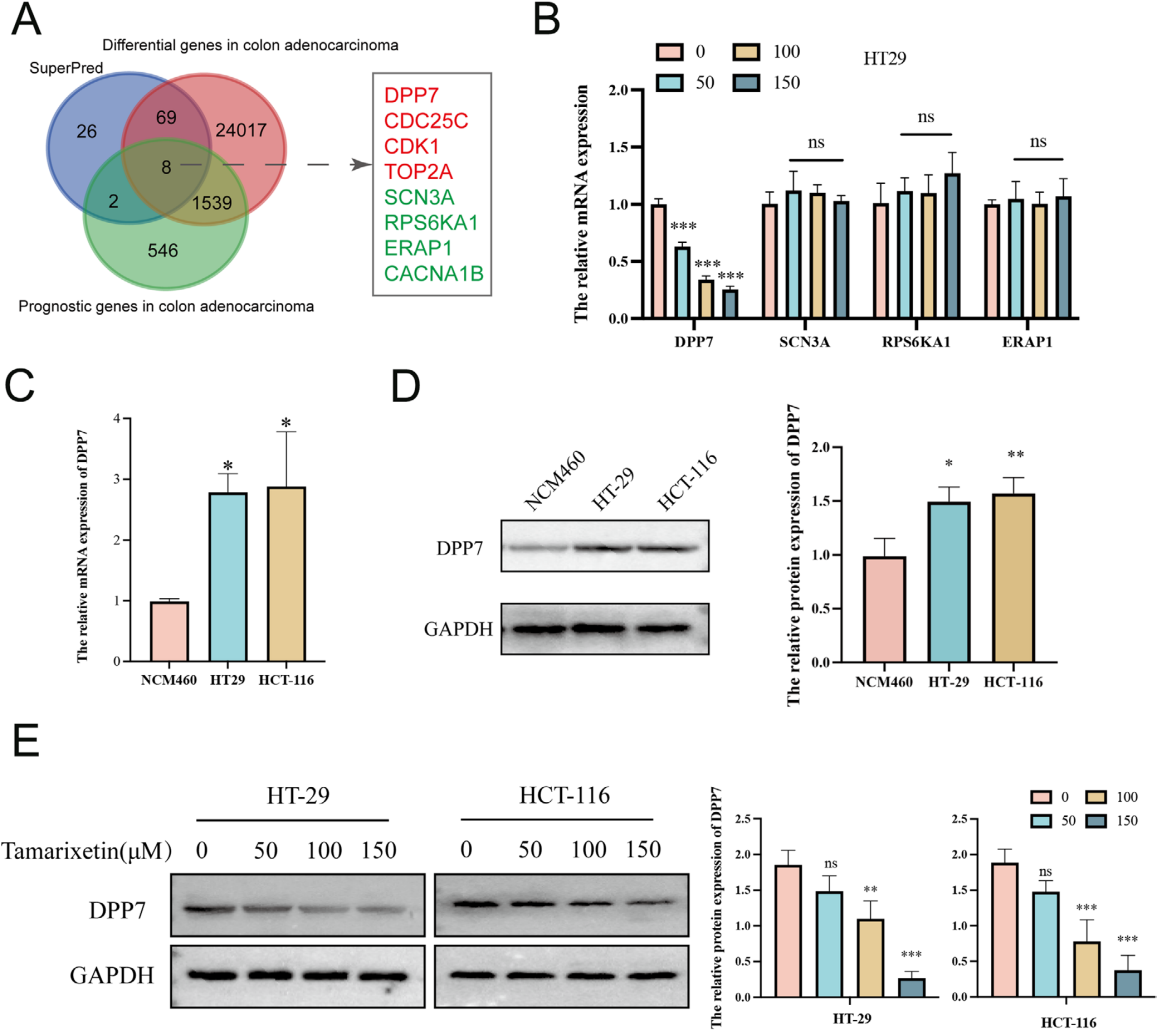
Tamarixetin modulates the progression of CRC through regulation of the DPP7. (A) Venn diagram depicting the intersection of potential target genes of Tamarixetin from the SuperPred database, differentially expressed genes between CRC and normal tissues from the TCGA‐COAD database, and prognosis‐related genes in CRC from the TCGA dataset. (B) qPCR analysis of four identified genes. (C) qPCR analysis of DPP7 mRNA expression in NCM460, HT‐29, and HCT‐116 cell lines. (D) Western blot analysis of the expression of DPP7 protein in NCM460, HT‐29 and HCT‐116 cell lines. (E) The effect of Tamarixetin on the protein level of DPP7 in CRC cells, GAPDH was used as a loading control, *N* = 3. *p < 0.05; **p < 0.01; ***p < 0.001 versus non‐treated cells.

To explore the mechanisms of the inhibitory effect of Tamarixetin on CRC, we first conducted a systematic exploration of the SuperPred database (Table S2). Then, an in‐depth analysis of gene expression differences between CRC tissues and normal tissues was performed, and prognosis‐related genes in CRC patients in the TCGA‐COAD database were further explored (Tables S3 and S4). Genes including DPP7, CDC25C, CDK1, TOP2A, SCN3A, RPS6KA1, ERAP1 and CACNA1B were found in all these three datasets (Figure 3A). Moreover, prognostic analysis indicated that the levels of DPP7, CDC25C, CDK1 and TOP2A were strongly associated with poor survival in patients (Figure S2A). Conversely, higher levels of SCN3A, RPS6KA1, ERAP1 and CACNA1B indicated a better prognosis in patients (Figure S2A). We further examined the mRNA levels of these genes in CRC cells and found DPP7 mRNA levels were decreased after Tamarixetin treatment (Figure 3B and Figure S2B). The mRNA and protein levels of DPP7 were higher in the HT‐29 and HCT‐116 cell lines compared to NCM460 (Figure 3C,D). The treatment of Tamarixetin significantly inhibited the protein levels of DPP7 in CRC cells (Figure 3E). These results indicate that Tamarixetin inhibits DPP7 expression in CRC cells.

### 3.4 DPP7 Mediates the Inhibitory Effect of Tamarixetin on CRC Cells

**FIGURE 4:**
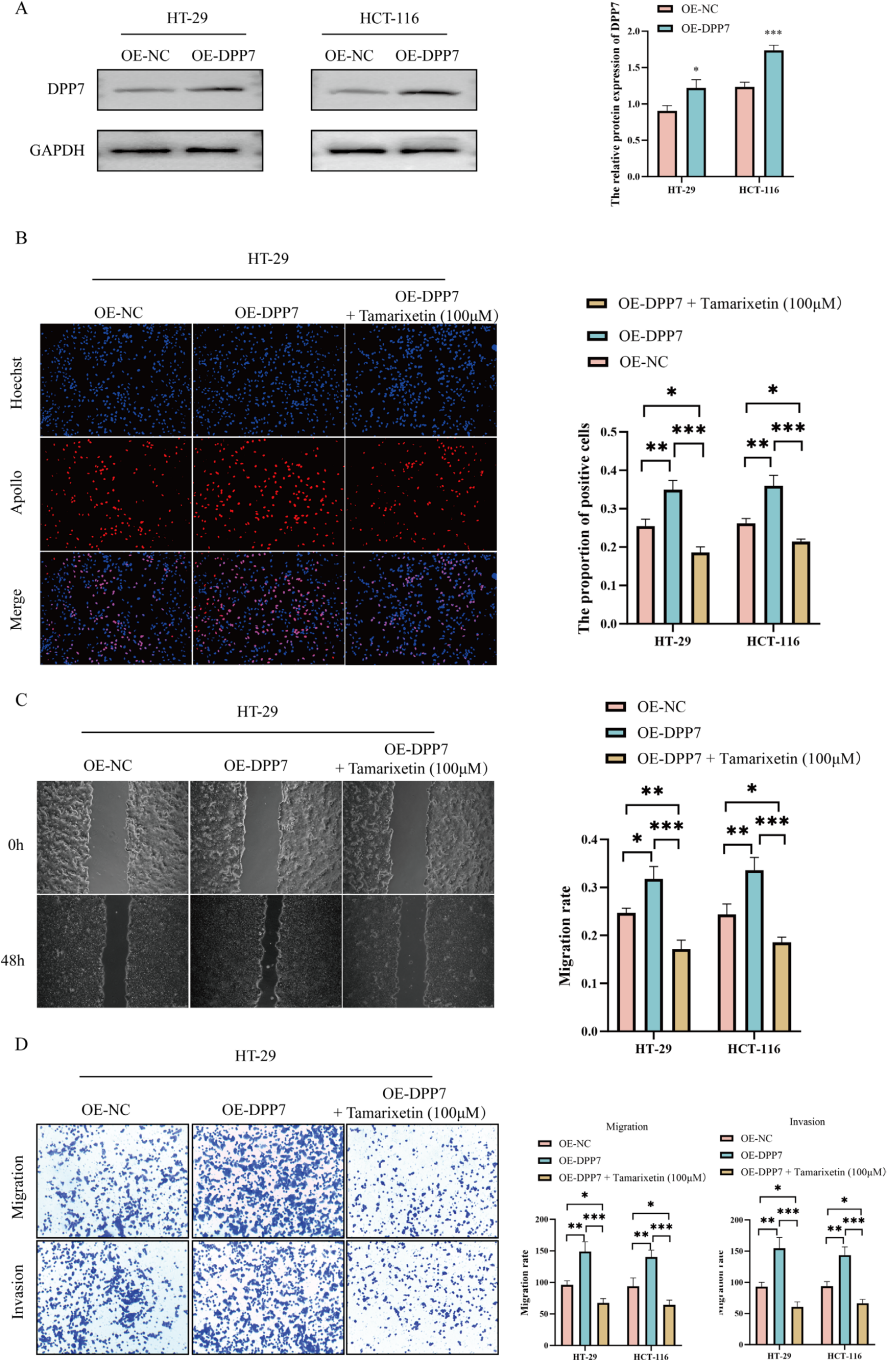
DPP7 overexpression promotes CRC progression. (A) Transfection efficiency was detected by Western blot. (B) HT‐29 and HCT‐116 cells were DPP7 overexpressed or DPP7 overexpressed while treated with Tamarixetin (100 μM) for 24 h, and then EdU assay was used to detect cell proliferation. (C, D) The effect of DPP7 overexpression or DPP7 overexpression while treated with Tamarixetin (100 μM) for 24 h on the migration and invasion of CRC cells based on wound healing assay (C) and transwell assay (D), *N* = 3. *p < 0.05; **p < 0.01; ***p < 0.001 versus non‐treated cells.

**FIGURE 5:**
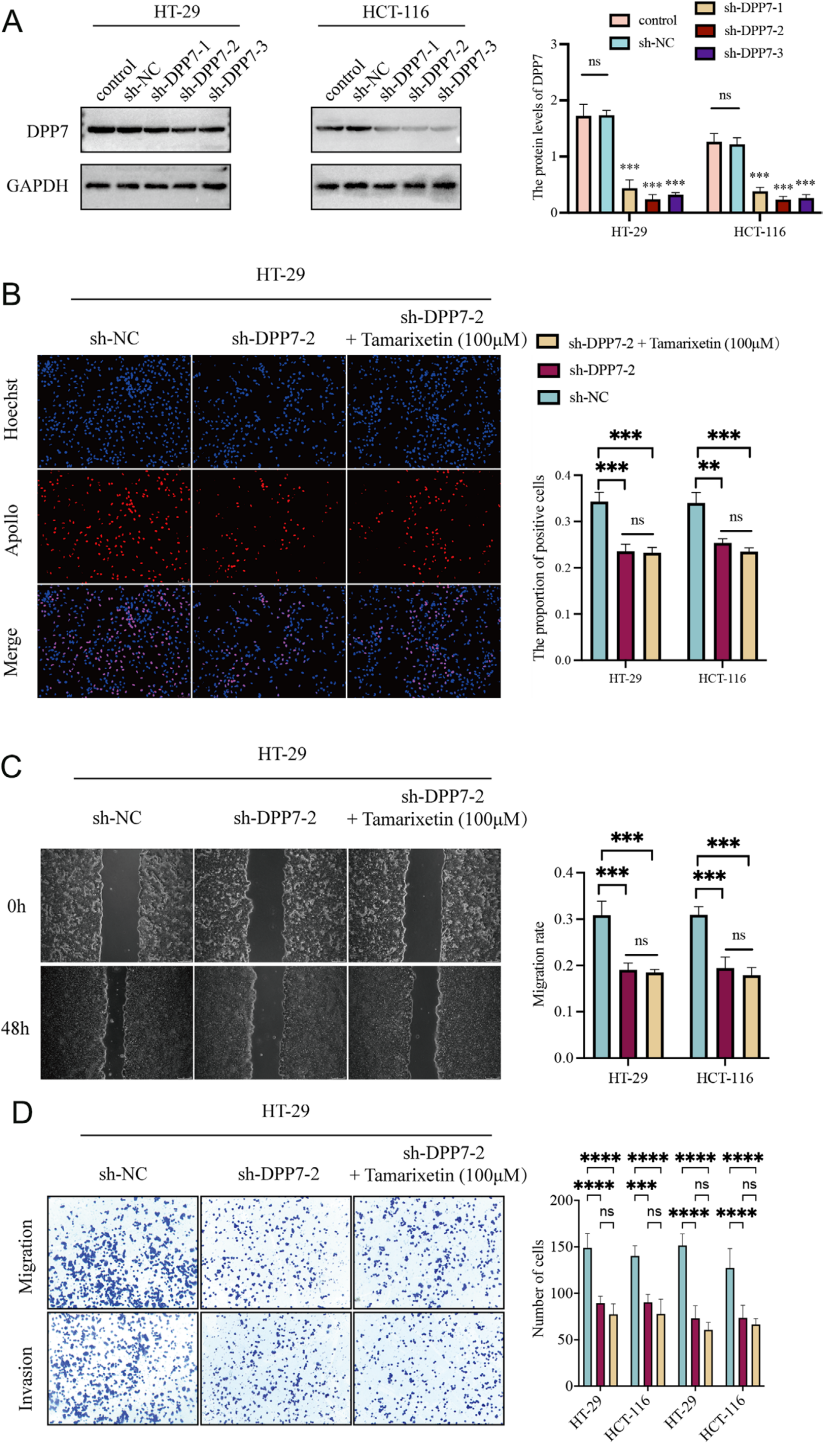
DPP7 silencing inhibits CRC progression. (A) Knock‐down efficiency was detected by Western blot. (B) HT‐29 and HCT‐116 cells were DPP7 silence or DPP7 silence while treated with Tamarixetin (100 μM) for 24 h, and then EdU assay was used to detect cell proliferation. (C, D) The effect of DPP7 silence or DPP7 silence while treated with Tamarixetin (100 μM) for 24 h on the migration and invasion of CRC cells based on wound healing assay and transwell assay, *N* = 3. *p < 0.05; **p < 0.01; ***p < 0.001 versus non‐treated cells.

To understand whether Tamarixetin suppresses proliferation, invasion, and migration of CRC cells via DPP7, we first overexpressed DPP7 in both CRC cell lines (Figure 4A). The overexpression of DPP7 significantly increased the number of proliferating cells (Figures 4B and S3A). Meanwhile, the overexpression of DPP7 effectively enhanced cell migration rates (Figures 4C and S3B) and increased the number of cells in the lower chamber of the transwell (Figures 4D and S3C). However, the treatment with Tamarixetin decreased the proliferation, migration, and invasion in these DPP7 overexpressing cells (Figure 4B–D). On the other hand, DPP7 was knocked down in CRC cells (Figure 5A) and the suppression of DPP7 resulted in lower EdU‐positive cell numbers and a significant reduction in migration and invasion capabilities in CRC cells (Figures 5B–D and S4A–C). Additionally, the treatment of Tamarixetin did not further inhibit the proliferation, migration, or invasion capacities in the DPP7‐knockdown cells (Figure 5B–D). These data suggest that Tamarixetin specifically targets DPP7, reducing CRC cell proliferation, invasion and migration.

### 3.5 Tamarixetin Exerts Anti‐Tumour Effect in CRC Cells via the Suppression of DPP7/WNT3A/β‐Catenin Pathway

**FIGURE 6:**
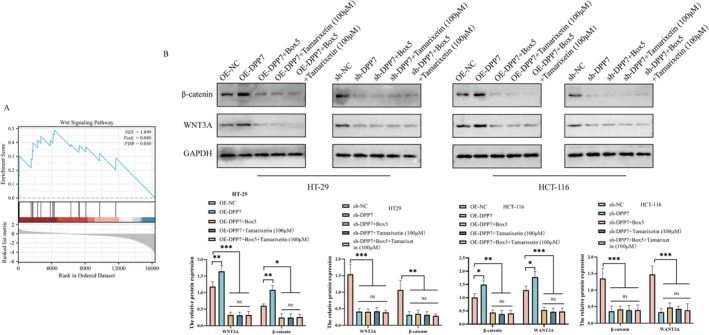
Tamarixetin promotes CRC progression via DPP7‐mediated activation of WNT3A/β‐catenin pathway. (A) GSEA revealing a strong association between DPP7 expression and the activation of the WNT signalling pathway in CRC samples. (B) Western blot analysis was utilised to assess the impact of DPP7 overexpression or silencing on the WNT signalling pathway. Subsequent treatments with Box5, Tamarixetin and a combination of both were also evaluated for their effects on the WNT pathway in the context of DPP7 modulation, GAPDH was used as a loading control, *N* = 3. *p < 0.05; **p < 0.01; ***p < 0.001 versus non‐treated cells.

To understand the potential mechanism that Tamarixetin inhibits CRC via DPP7 suppression, we performed GSEA analysis in the TCGA database and identified that DPP7 is closely associated with the activation of the WNT signalling pathway in CRC tumours (Figure 6A). The inhibition of DPP7 significantly decreased the protein levels of β‐catenin and WNT3A, whereas the overexpression of DPP7 increased the levels of these proteins (Figure 6B). Next, we treated the DPP7 overexpressing cells with Tamarixetin and/or Box5 (a WNT pathway inhibitor). The protein levels of β‐catenin and WNT3A were significantly decreased in DPP7 overexpressing CRC cells with the treatment of Tamarixetin or Box5. Notably, the levels of β‐catenin and WNT3A were not further reduced with the combination of Tamarixetin and Box5 (Figure 6B). Moreover, the protein levels of β‐catenin and WNT3A showed no significant difference in DPP7 knockdown cells with the treatment of Tamarixetin and/or Box5 (Figure 6B).

To validate whether DPP7/WNT3A/β‐catenin pathway is essential for the anti‐tumour effect of Tamarixetin, we examined the proliferation of DPP7 overexpressing cells after the treatments. Treatment with Tamarixetin and BOX5 decreased the proliferation of CRC cells overexpressing DPP7, but there was no significant difference in the inhibition rate among the treatment groups (Figure S5A). Also, the proliferation of DPP7 knockdown cells remains similar upon the treatment of Tamarixetin and/or BOX5 (Figure S5A). The DPP7 overexpressing cells showed lower percentages of migration and invasion, while the DPP7 knockdown ones were not affected by the treatment of Tamarixetin and/or BOX5 (Figure S5B,C). Together, these findings show that the sensitivity of CRC cells to Tamarixetin depends on the DPP7/WNT3A/β‐catenin signalling pathway.

## 4 Discussion

In colorectal cancer cases facing recurrence and drug resistance, the need for novel anticancer strategies has been critical [14, 15, 16]. Natural compounds have been reported to suppress tumorigenesis and apply as potential anti‐cancer candidates, but their lethal effects and underlying anti‐cancer mechanisms remain to be explored. Tamarixetin, a natural flavonoid, has shown antitumor properties across several types of tumours [17, 18], and it has been reported that tamarixetin hinders nuclear translocation as well as the activity of NF‐kB in various solid cancers including CRC, demonstrating its anti‐invasive potential [17]. However, its efficacy in CRC remains unknown. Given that NF‐kB is activated in stromal myofibroblasts surrounding colon adenocarcinomas promoting migration and invasion of CRC cells [19], it has been suggested that there is potential use of tamarixetin as a template for novel therapeutic applications toward treatment and/or prevention of CRC. In our study, we evaluated the application of tamarixetin on colorectal cell lines and patient‐derived organoids. We discovered that tamarixetin has limited cellular toxicity on normal colorectal epithelial cell lines and normal tissues in nude mice, but significant suppression of CRC cell proliferation, invasion, and migration. Moreover, Tamarixetin inhibited the growth of patient‐derived CRC organoids in vitro. These results demonstrated Tamarixetin's potent anti‐cancer properties.

On the other hand, we also identified dipeptidyl peptidase 7 (DPP7) as a target of Tamarixetin. DPP7 is a glycoprotein that is sequestered in intracellular vesicles and is distinct from the lysozymes. It is secreted in its active form during the release of calcium [20]. The enzyme's catalytic activity necessitates homodimerization via a leucine zipper motif and exhibits functionality across a broad pH range, with optimal activity between pH 5.5 and 7.0 [21, 22, 23]. DPP7 is involved in the G_0_ survival program of lymphocytes and neuronal cells, with its inhibition leading to the apoptosis of these quiescent cells [24]. Furthermore, DPP7 contributes to the pathogenesis of B cell chronic lymphocytic leukaemia (B‐CLL), as B cells arrest in G_0_ accumulate in the peripheral blood of CLL patients, making susceptibility to DPP7‐induced apoptosis a prognostic marker for CLL outcomes [25]. The natural substrates of DPP7 remain unidentified. This study focuses specifically on the role of DPP7 in colorectal cancer. It has been found that overexpression of DPP7 counteracts the suppressive effect of Tamarixetin on colorectal cancer. This suggests that DPP7 plays a pivotal role in the progression of colorectal cancer, which underlines the therapeutic potential of Tamarixetin by inhibiting DPP7.

The Wnt signalling cascade plays a pivotal role in a myriad of biological processes, encompassing embryonic development, cell cycle regulation, inflammation, and carcinogenesis [26]. The proteins of the Wnt pathway are recognised not only as reliable biomarkers but also as promising therapeutic targets in cancer management [27, 28]. Wnt signal transduction is divided into a canonical and a non‐canonical pathway. These pathways are crucial for cell survival, proliferation, and differentiation as well as for cell polarity and migration [29]. This study focuses on the role of DPP7 in CRC. We found that overexpression of DPP7 activates the Wnt signalling pathway and promotes the progression of CRC. Furthermore, an inhibitor of the Wnt pathway showed to be lethal for colorectal cancer cells overexpressing DPP7. This indicates that DPP7 is responsible for the development of CRC, demonstrating that the lethal effect of Tamarixetin on CRC cells is through the inhibition of DPP7.

In summary, our study reveals that Tamarixetin holds promise in suppressing colorectal cancer cells but not normal tissues. By specifically targeting DPP7 and disrupting the WNT3A/β‐catenin signalling pathway, Tamarixetin offers a potential strategy for precision oncology in CRC. Further investigations are required to explore the clinical potential of Tamarixetin as a therapeutic agent and to evaluate the efficacy and safety in clinical settings.

## Author Contributions

**Peng Ouyang:** conceptualization (equal), data curation (equal), formal analysis (equal), methodology (equal), validation (equal), writing – original draft (equal), writing – review and editing (equal). **Jin Gong:** conceptualization (equal), formal analysis (equal), validation (equal), writing – original draft (equal), writing – review and editing (equal). **Jinlin Nie:** conceptualization (equal), formal analysis (equal), methodology (equal), supervision (equal), writing – original draft (equal), writing – review and editing (equal). **Sridhar Kandala:** data curation (equal), formal analysis (equal), methodology (equal), writing – original draft (equal). **Yangdong Shi:** data curation (equal), formal analysis (equal), methodology (equal), writing – original draft (equal). **Yao Tian:** data curation (equal), formal analysis (equal), methodology (equal), writing – original draft (equal). **Zhijing Zhang:** writing – original draft (equal), writing – review and editing (equal). **Sifu Fang:** writing – review and editing (equal). **Fan Pan:** conceptualization (equal), formal analysis (equal), funding acquisition (equal), methodology (equal), supervision (equal), writing – original draft (equal), writing – review and editing (equal). **Lin Qiu:** conceptualization (equal), formal analysis (equal), project administration (equal), supervision (equal), writing – original draft (equal), writing – review and editing (equal). **Zhen Bao:** funding acquisition (lead), supervision (equal), writing – original draft (equal), writing – review and editing (equal).

## Ethics Statement

All experiments were performed in compliance with the relevant regulations, and all patients provided written informed consent. The ethical approval for our research was granted by The First Affiliated Hospital of Jinan University (KY‐2023‐325). Besides, the animal studies were approved by the Animal Ethics Committee of Jinan University (Approval No. IACUC‐20240322‐06). The experiments followed the Guidelines for the Care and Use of Laboratory Animals issued by the Chinese Council on Animal Research.

## Consent

All the authors agree to the content of the paper.

## Conflicts of Interest

The authors declare no conflicts of interest.

## Supporting information


**Figure S1.** Tamarixetin promotes CRC progression via DPP7‐mediated activation of WNT3A/β‐catenin pathway. (A) EdU was utilised to assess the impact of DPP7 overexpression or silencing on CRC cell proliferation. Subsequent treatments with Box5, Tamarixetin, and a combination of both were also evaluated for their effects on the proliferation ability in the context of DPP7 modulation, *N* = 3; (B) Wound‐healing was utilised to assess the impact of DPP7 overexpression or silencing on CRC cell migration. Subsequent treatments with Box5, Tamarixetin, and a combination of both were also evaluated for their effects on the migration ability in the context of DPP7 modulation, *N* = 3; (C) Transwell was utilised to assess the impact of DPP7 overexpression or silencing on CRC cell migration and invasion. Subsequent treatments with Box5, Tamarixetin, and a combination of both were also evaluated for their effects on the migration and invasion ability in the context of DPP7 modulation, *N* = 3. *p < 0.05; **p < 0.01; ***p < 0.001 versus non‐treated cells.

**Figure S2.** Tamarixetin affects the prognosis of CRC via suppressing the expression of DPP7. (A) Kaplan–Meier survival curves illustrating the prognostic significance of the eight identified genes (Red line: High expression; Cyan line: Low expression). The dotted line indicates median survival; (B) qPCR analysis four identified genes.

**Figure S3.** DPP7 overexpression promotes CRC progression in HCT‐116. (A) HCT‐116 cells were DPP7 overexpression or DPP7 overexpression while treated with Tamarixetin (100 μM) for 24 h, and then EdU assay was used to detect cell proliferation; (B‐C) The effect of DPP7 overexpression or DPP7 overexpression while treated with Tamarixetin (100 μM) for 24 h on the migration and invasion of CRC cells based on wound healing and transwell assays.

**Figure S4.** DPP7 silencing inhibits CRC progression in HCT‐116. (A) HCT‐116 cells were DPP7 silence or DPP7 silence while treated with Tamarixetin (100 μM) for 24 h, and then EdU assay was used to detect cell proliferation; (B‐C) The effect of DPP7 silence or DPP7 silence while treated with Tamarixetin (100 μM) for 24 h on the migration and invasion of CRC cells based on wound healing and transwell assays.

**Figure S5.** Tamarixetin promotes CRC cell proliferation, migration, and invasion via DPP7‐mediated activation of WNT3A/β‐catenin pathway. (A) EdU assay was used to detect cell proliferation under different treatment in HT‐29 (Left) and HCT‐116 (Right); (B‐C) The migration and invasion of HT‐29 (Left) and HCT‐116 (Right) under different treatment based on wound healing and transwell assays, *N* = 3. *p < 0.05; **p < 0.01; ***p < 0.001.

**Table S1.** Supporting Information.

**Table S2.** Supporting Information.

**Table S3.** Supporting Information.

**Table S4.** Supporting Information.

**Table S5.** Supporting Information.

## Data Availability

The authors have nothing to report.
