# Supplementary figures and images for: Tamarixetin Suppresses Colorectal Cancer Progression by Targeting DPP7‐Mediated WNT3A/β‐Catenin Signalling Pathway
Source: J Cell Mol Med. 2025 Aug 22;29(16):e70787. doi: 10.1111/jcmm.70787 (PMC12372979; doi:10.1111/jcmm.70787)

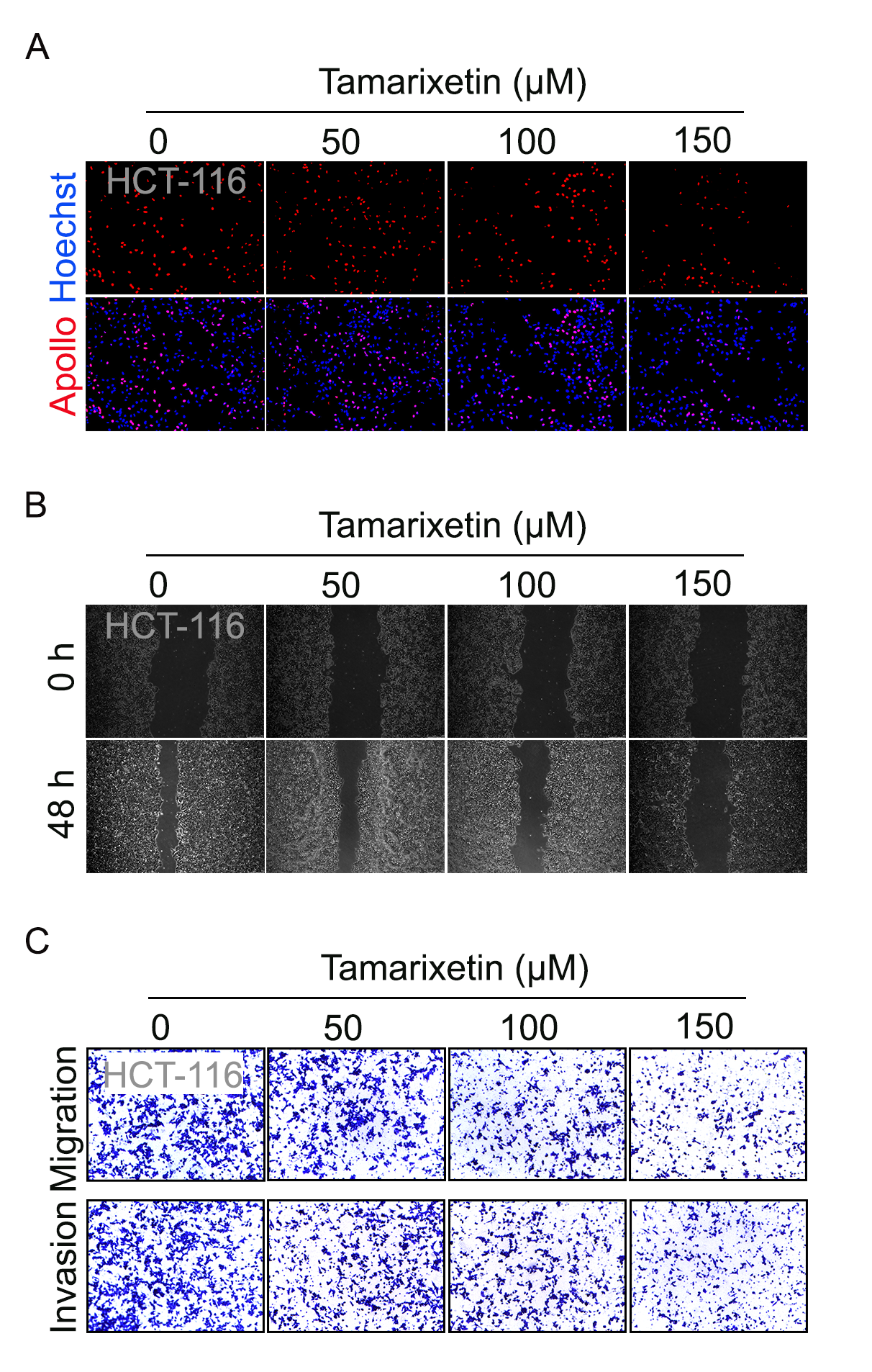

Supplement: Supplementary file 1 — Figure S1. Tamarixetin promotes CRC progression via DPP7‐mediated activation of WNT3A/β‐catenin pathway. (A) EdU was utilised to assess the impact of DPP7 overexpression or silencing on CRC cell proliferation. Subsequent treatments with Box5, Tamarixetin, and a combination of both were also evaluated for their effects on the proliferation ability in the context of DPP7 modulation, N = 3; (B) Wound‐healing was utilised to assess the impact of DPP7 overexpression or silencing on CRC cell migration. Subsequent treatments with Box5, Tamarixetin, and a combination of both were also evaluated for their effects on the migration ability in the context of DPP7 modulation, N = 3; (C) Transwell was utilised to assess the impact of DPP7 overexpression or silencing on CRC cell migration and invasion. Subsequent treatments with Box5, Tamarixetin, and a combination of both were also evaluated for their effects on the migration and invasion ability in the context of DPP7 modulation, N = 3. *p < 0.05; **p < 0.01; ***p < 0.001 versus non‐treated cells. [file JCMM-29-e70787-s002.tif]

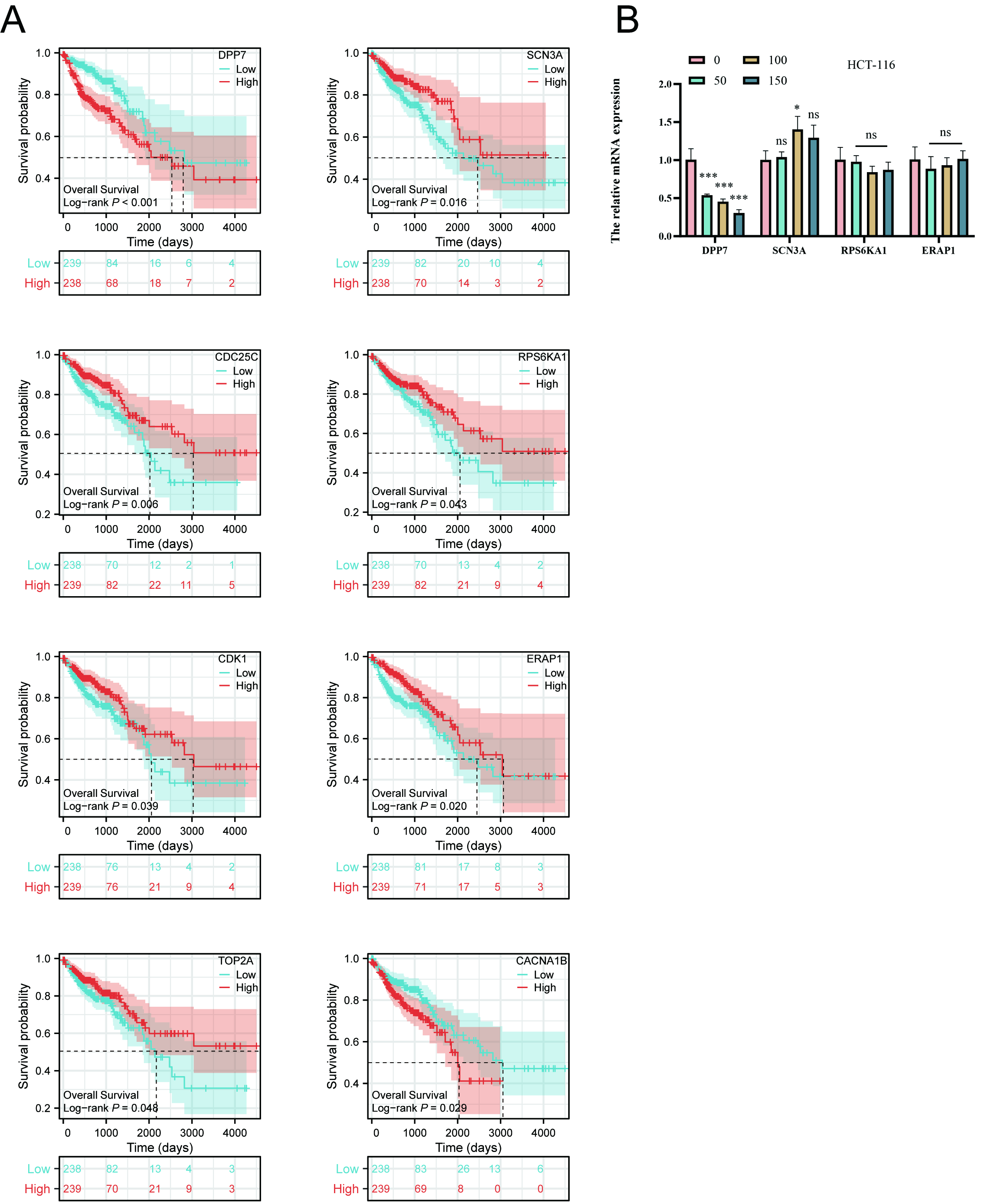

Supplement: Supplementary file 2 — Figure S2. Tamarixetin affects the prognosis of CRC via suppressing the expression of DPP7. (A) Kaplan–Meier survival curves illustrating the prognostic significance of the eight identified genes (Red line: High expression; Cyan line: Low expression). The dotted line indicates median survival; (B) qPCR analysis four identified genes. [file JCMM-29-e70787-s001.tif]

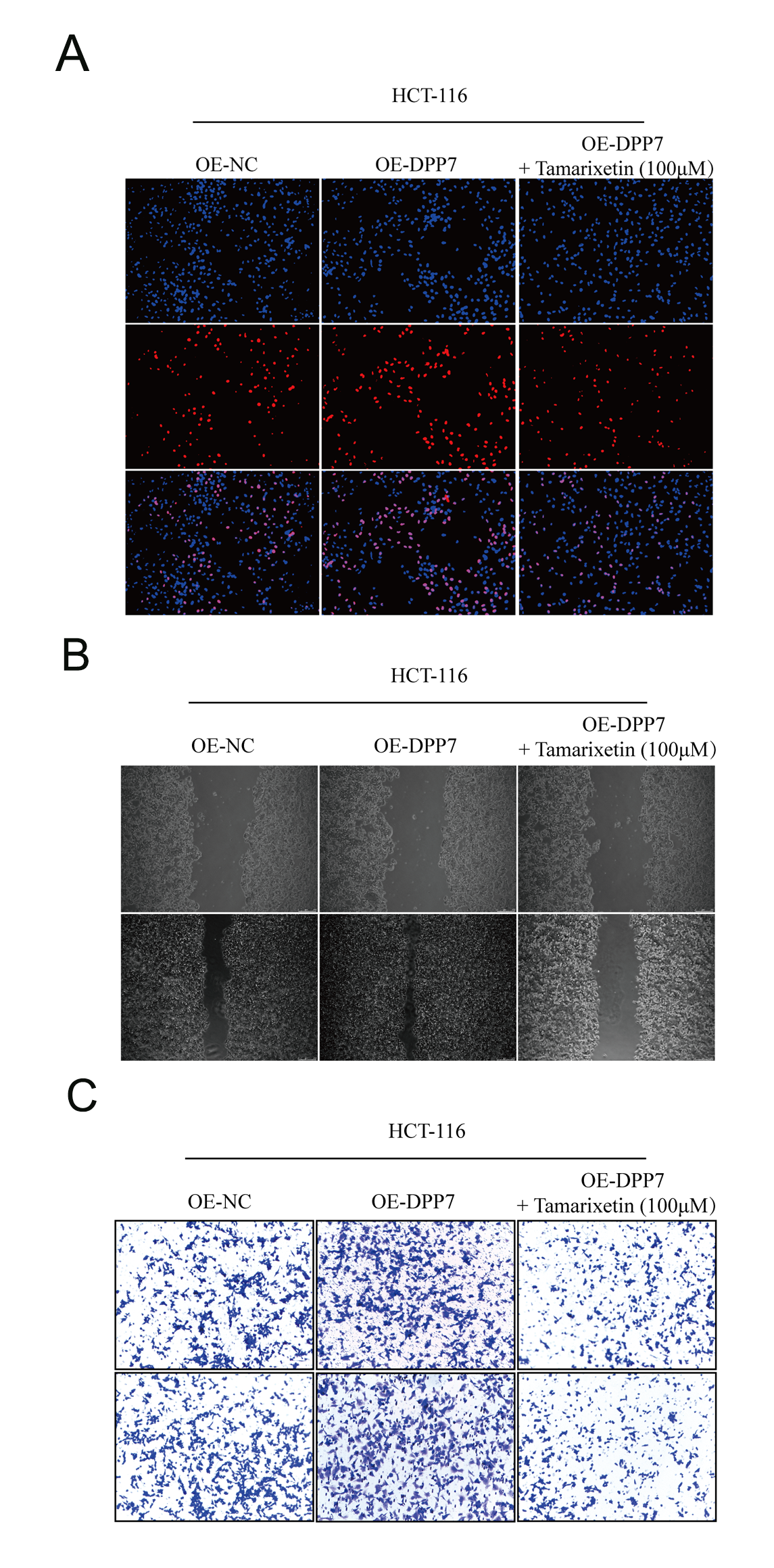

Supplement: Supplementary file 3 — Figure S3. DPP7 overexpression promotes CRC progression in HCT‐116. (A) HCT‐116 cells were DPP7 overexpression or DPP7 overexpression while treated with Tamarixetin (100 μM) for 24 h, and then EdU assay was used to detect cell proliferation; (B‐C) The effect of DPP7 overexpression or DPP7 overexpression while treated with Tamarixetin (100 μM) for 24 h on the migration and invasion of CRC cells based on wound healing and transwell assays. [file JCMM-29-e70787-s006.tif]

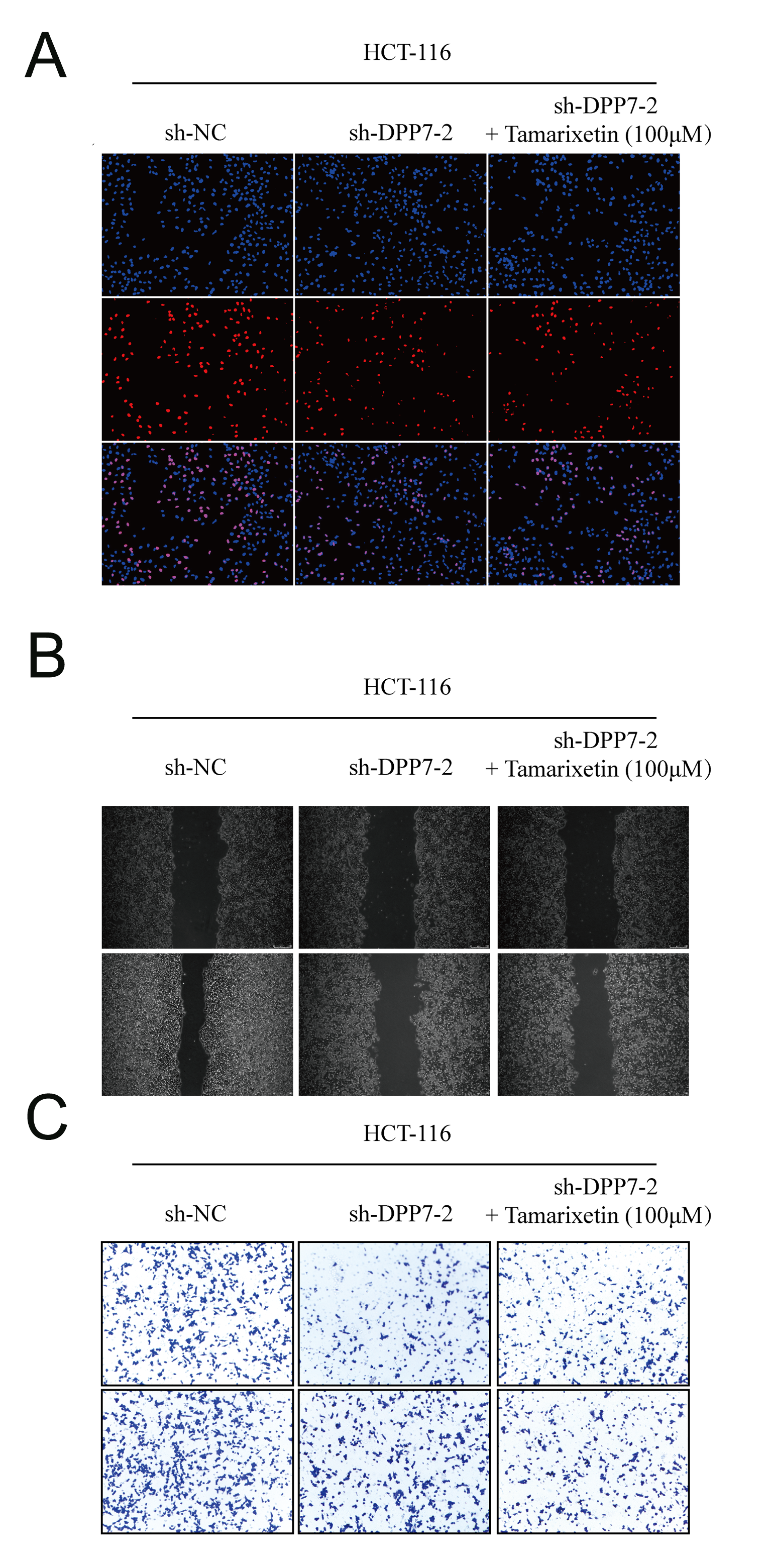

Supplement: Supplementary file 4 — Figure S4. DPP7 silencing inhibits CRC progression in HCT‐116. (A) HCT‐116 cells were DPP7 silence or DPP7 silence while treated with Tamarixetin (100 μM) for 24 h, and then EdU assay was used to detect cell proliferation; (B‐C) The effect of DPP7 silence or DPP7 silence while treated with Tamarixetin (100 μM) for 24 h on the migration and invasion of CRC cells based on wound healing and transwell assays. [file JCMM-29-e70787-s009.tif]

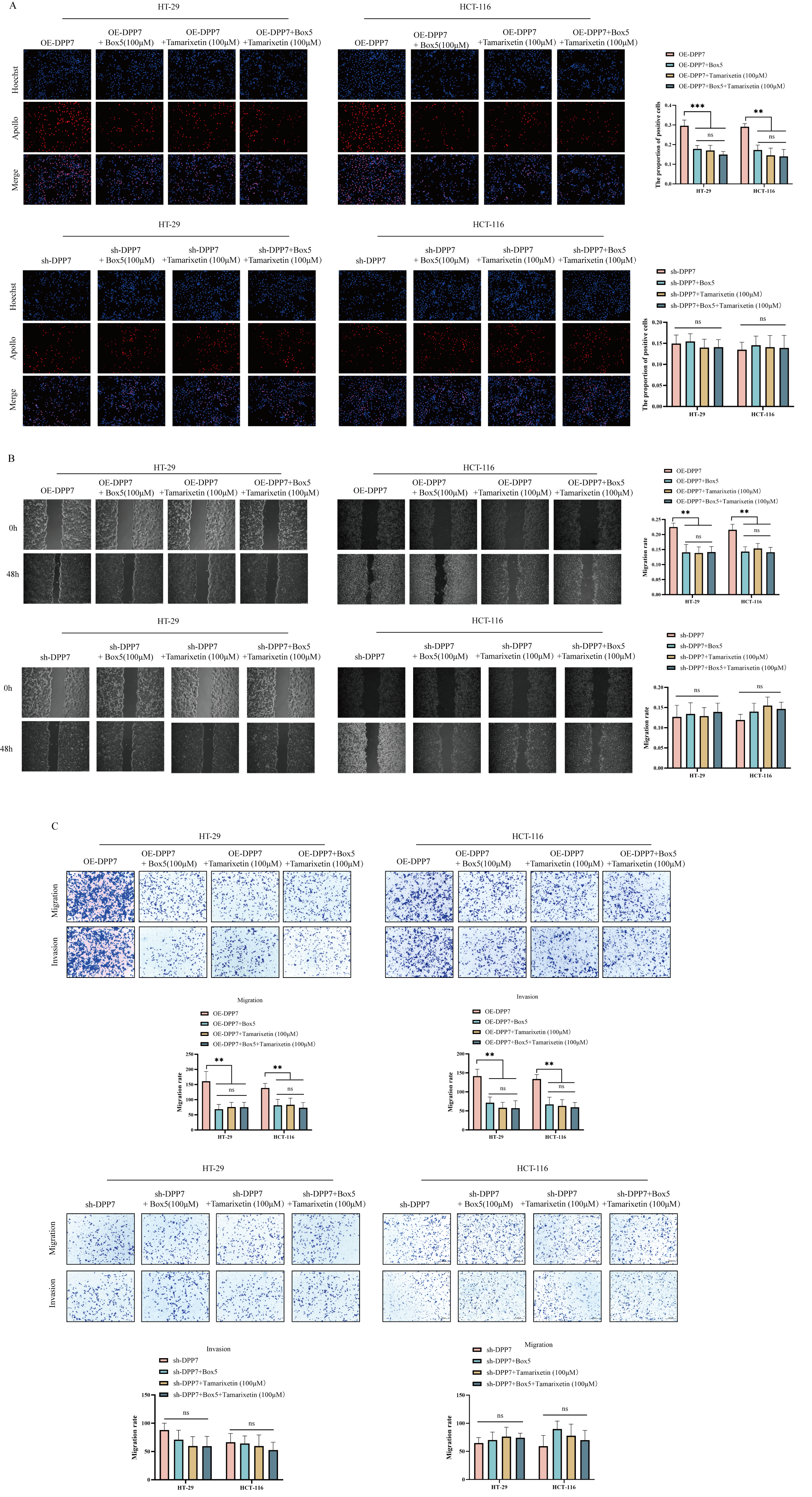

Supplement: Supplementary file 5 — Figure S5. Tamarixetin promotes CRC cell proliferation, migration, and invasion via DPP7‐mediated activation of WNT3A/β‐catenin pathway. (A) EdU assay was used to detect cell proliferation under different treatment in HT‐29 (Left) and HCT‐116 (Right); (B‐C) The migration and invasion of HT‐29 (Left) and HCT‐116 (Right) under different treatment based on wound healing and transwell assays, N = 3. *p < 0.05; **p < 0.01; ***p < 0.001. [file JCMM-29-e70787-s005.tif]
